# Site readiness practices for clinical trials – considerations for CTSA hubs

**DOI:** 10.1017/cts.2023.569

**Published:** 2023-06-29

**Authors:** Laura Viera, Laura James, Anantha Shekhar, Octavian C. Ioachimescu, John B. Buse

**Affiliations:** 1 University of North Carolina School of Medicine, Chapel Hill, NC, USA; 2 University of Arkansas for Medical Sciences, Little Rock, AR, USA; 3 University of Pittsburgh, Pittsburgh, PA, USA; 4 Medical College of Wisconsin, Milwaukee, WI, USA

**Keywords:** CTSA, site readiness practices, clinical trials, operations

It has been widely recognized that site-specific attributes such as infrastructure, personnel, and participant recruitment-related factors are critical to effective and successful conduct of clinical trials [1–4]. Industry, federal, and foundation sponsors consider these factors in their assessment of site readiness when selecting sites to execute clinical trial protocols. While site selection factors vary somewhat depending on sponsor and trial type, “A Framework for Assessing for Clinical Trial Site Readiness” has identified a core set of site readiness practices that are recommended for all sites interested in initiating and executing clinical trials [5]. The site readiness practices include factors spanning domains of research team, infrastructure, study management, data collection and management, quality oversight, and ethics and safety (Table 1).

**Table 1.** List of site readiness practices for clinical trials, organized by domain

Domain	Site readiness practices
Research team	The research team has sufficient and diverse personnel, to support the roles and functions needed to conduct a clinical trial and enroll trial participants who accurately reflect the patient population for the disease or condition being studied, with particular consideration for underrepresented and underserved groups. The Principal Investigator is qualified through experience, training, and mentorship to lead and conduct clinical trials and is free from regulatory debarment and other disciplinary actions that would prevent them from practicing medicine and conducting clinical research. Sub-investigators and other research team members are qualified through experience, training, and mentorship to conduct clinical trials, are well trained in cultural humility and strategies for engaging with underrepresented communities, and free from disciplinary actions that would prevent them from conducting clinical trials. All research team members receive initial and refresher training to perform clinical trial activities per ICH GCP standards, and as appropriate, have received training that is tailored to an individual’s role and specific to the study protocol.
Infrastructure	Identify all satellite sites, external and community facilities, and contractors utilized to fulfill the requirements of studies. Ensure facilities (including satellite sites, external facilities, and contractors) and equipment are adequate to fulfill the requirements of a study. Provide reliable physical and operational infrastructure (e.g., electric power, internet access, telephone, email, and communications). Along with community affiliates, store documents, materials, product, and equipment in a secure location protected against theft, damage, tampering, or other harms during the duration of a study. Retain study records after the conclusion of a study pursuant to national, state, local, and other applicable requirements and study protocol. Safeguard staff and participants and secure virtual and physical assets (e.g., facilities, records, specimens) during a disruption of operations (e.g., natural disaster). Maintain essential functions after a major disruption of operations (e.g., natural disaster). Protect computers, networks, programs, and data from digital disruptions and attacks. Maintain interoperable information systems and technology capabilities (e.g. data standards, quality control), adequate to support clinical trial conduct. Initiate study (e.g., execute a contract) in a prompt manner. Ensure sufficient processes for hiring and supporting diverse staff to fulfill the roles and functions needed to conduct a clinical trial (e.g., Principal/sub investigators, clinical research associates, research nurses, data managers, and study coordinators). Identify and manage conflicts of interest, including complete financial disclosures for research team members, pursuant to national, state, local, and other applicable requirements and study protocol.
Study management	Research team utilizes standard operating procedures/processes for the conduct of clinical trials pursuant to national, state, local, and other applicable requirements and study protocol. Principal investigator monitors and can demonstrate oversight for all study-related activities, including those functions delegated to satellite sites and contractors, including recruitment, enrollment, and retention suitable for reflecting the diversity of the populations affected by the disease or intervention of study. Research team can execute study initiation, start-up, and close-out procedures in a prompt manner. Research team has access to and process for recruiting and retaining eligible study participants, which should include a plan for enrolling adequate numbers of participants from populations that are underrepresented and underserved in clinical trials. Research team can collect, handle, label, store, and ship digital and biological samples (e.g., cultures, blood, serum, plasma, urine, feces, tissues, imaging) with appropriate documentation pursuant to national, state, local, and other applicable requirements and study protocol. Research team can handle investigational medical products, devices, and other means of intervention safely and securely and can record receipt, expiry, reconstitution, handling, dispensation, transfer, and/or destruction. Research team can establish, maintain, and record calibration for study-specific equipment. Research team can maintain essential study documentation before, during, and after a trial. Responsible party^2^ must report study results to clinicaltrials.gov within the required times before, during, and after the conclusion of a study, and has a strategy for dissemination of research findings to stakeholders and participants.
Data collection and management	Research team implements controls (e.g., audits, system validations, audit trails, electronic signatures, and documentation) for software and systems involved in processing study-related data pursuant to national, state, local, and other applicable requirements and study protocol. Research team can collect, access, retrieve, and exchange data in a timely, accurate, and complete manner. Monitors, sponsor personnel, and regulatory authorities have access to source material, electronic data systems, facilities, and source documents. Research team can ensure quality control of data and source documentation to ensure the integrity and proper reporting of study data. Research team can ensure blinding/masking, while promoting transparency and trust with study participants regarding how their data will be used and who will have access to it.
Quality oversight	Research team can ensure and verify that the quality requirements have been fulfilled pursuant to national, state, local, and other applicable requirements and study protocol. Research team members are able and empowered to identify, prevent, report, and correct safety and quality issues in a timely fashion. Research team can identify and remediate deficiency findings from regulatory inspections and sponsor audits (e.g., warning letters, FDA Form 483, corrective and preventive action).
Ethics and safety	Research team can protect the rights and welfare of trial participants pursuant to national, state, local, and other applicable requirements and study protocol. Research team can identify, assess, process, and report safety events (e.g., deviations, malfunctions, deficiencies, adverse events) pursuant to national, state, local, and other applicable requirements and study protocol. Research team can execute an informed consent/assent process that is respectful of participants and pursuant to national, state, local, and other applicable requirements and study protocol. Research team can maintain confidentiality for study participants, while promoting transparency and trust with participants regarding how their data will be used and who will have access to it. Research team has access to and reports to a properly constituted IRB/ethics committee pursuant to national, state, local, and other applicable requirements and study protocol. Research team engages with study participants, especially vulnerable populations (e.g., children, refugees, people with an intellectual or developmental disability) and populations that have experienced medical abuse and exploitation (e.g., racial and ethnic minorities), in an ethical and culturally appropriate manner, and addresses institutional racism through intentional recruitment and engagement strategies. Research team clearly communicates study risks and benefits to study participants in a manner that is accessible and culturally/linguistically appropriate.

Adoption of site readiness practices aims to improve sites’ success in initiating and sustaining clinical trials and streamlining operations. Nevertheless, there are several potential challenges sites could face in implementing the site readiness practices. With robust centralized infrastructure and sophisticated personnel expertise, Clinical and Translational Science Awards (CTSA) hubs are well-positioned to lead the adoption, documentation, maintenance, and further evaluation of site readiness practices. Furthermore, the framework defines three cross-cutting principles essential for site readiness: a culture of quality, clinical research literacy, and person-centeredness. The work of the CTSA hubs inherently encompasses these qualities, further priming them for leading these efforts in their institutions. In this paper, we highlight several anticipated challenges, opportunities, and recommendations to assist CTSA hubs in planning for and implementing site readiness practices for clinical trials.^1^

## Challenges and Opportunities for CTSA hubs

The process of standardizing operations as required to implement the site readiness practices will present challenges and is likely to face adoption delays and covert or frank opposition. Often, at large academic medical centers — where CTSA hubs are typically based — there is variability in standards and processes between various clinical units, departments, and locations. This variability is even greater in healthcare systems that often have multiple facilities and practices, sometimes with distinct governance. However, the CTSA hubs’ focus on innovation, efficiency, and quality, as well as experience in enabling enterprise-wide collaborations, allows them to understand existing differences in processes and to identify optimal local standards. Additionally, CTSA personnel are likely to be viewed as more neutral than personnel affiliated with a specific department or practice, and therefore potentially more successful in identifying suitable standards and subsequently implementing standardized processes that have historically been characterized by wider variability.

Implementing the site readiness practices may cause some disruption for site personnel as operations are standardized and processes change. CTSA hubs can minimize this disruption through their leadership across institutions, education, celebration of local successes, engagement of early adopters, and through the expansion and dissemination of existing resources or via training programs developed and vetted as part of the CTSA infrastructure.

The process of working across complex organizations to identify and define promising approaches to adoption will require significant and persistent effort. Simply documenting information from various units or locations will require coordinated effort, while working to establish (i.e., adopt, implement, maintain, and disseminate) new standards will require considerably more. CTSA hubs can leverage the advantage of existing personnel resources and technical infrastructure to refine approaches to adoption and document the site readiness practices in a centralized and accessible location, according to a set of defined organizational standards and principles of team science, including stakeholder engagement and shared decision-making.

## Recommendations for CTSA hubs

Most of the recommended site readiness practices are policies and standardized procedures based on regulatory requirements. Some of the site readiness practices likely already exist (e.g., record retention policies and disaster recovery plans), but may need to be harmonized across locations or units. Other site readiness practices may need to be established *de novo* (e.g., research teams’ utilization of standard operating procedures and adoption of prompt study initiation processes). Once identified or established, the site readiness practices will need to be documented and continually reviewed for appropriateness, accuracy and optimal use, especially given the rate of change across the national clinical research enterprise and local conditions. In order to realize optimal efficiency and ease in developing and maintaining site readiness practices, we make the following recommendations for CTSA hubs:

- Develop institutional standard operating procedures (SOPs) based on federal and local regulations according to the domains defined in the site readiness practices;
- Create a standardized template for work instructions that individual units or locations may implement to supplement institutional SOPs;
- Develop a robust SOP implementation plan that includes access to resources and trainings beneficial to study teams as they transition to new standard procedures;
- Establish a rigorous review schedule and plan to appropriately update and maintain the SOPs; and
- Harmonize documentation of and access to the site readiness practices across the institution.

Given that CTSA hubs are established and supported in various manners across institutions, some may be better-positioned to lead this charge. There are several factors that we believe will bolster the ability of CTSAs wishing to implement the site readiness practices across large and complex institutions:

- Close engagement with leadership of the University, healthcare system, and/or School of Medicine
- Alignment with institutional compliance offices (e.g. contracting, regulatory)
- Collaboration with the healthcare system, especially spanning distinct sites

## Call to Action

Implementation of the site readiness practices to demonstrate a site’s ability to conduct a clinical trial may be challenging for diverse and complex institutions. However, the anticipated benefits of increased engagement with clinical trial sponsors, greater trial opportunities for patients and communities, reduced burden for study teams related to initiating and executing clinical trials, and improved efficiency and quality are substantial.

Given the collaborative nature of the CTSA consortium and its rich expertise in all aspects of clinical trial design and conduct, implementation of the site readiness practices by CTSA hubs offers the unique opportunity of additional value and efficiency through sharing methods and feedback over time. With engagement from CTSA hubs, the site readiness practices may be further refined, and novel methods and processes for demonstrating and maintaining the readiness factors may be developed and shared across institutions.

## Footnotes

1 This article provides additional context and commentary for CTSA sites regarding the implementation of a set of site readiness practices identified in the article, “A Framework for Assessing Clinical Trial Site Readiness.” That article, which is cited in the references of this publication, describes the full set of site readiness practices, the methods for their selection, and a rationale for their use by clinical trial sites. The article also includes supplementary material, including guidance and resources for implementing and documenting the site readiness practices.

2 For more information on who qualifies as a responsible party, see https://www.ecfr.gov/current/title-42/chapter-I/subchapter-A/part-11 (accessed August 15, 2022).
